# Nano spray-dried powders for efficient pulmonary delivery of proteins: an emphasis on comparisons of derived metrics determined by chapters <601> and <1604>

**DOI:** 10.1080/10717544.2026.2732440

**Published:** 2026-09-15

**Authors:** Hao-Ying Li, Jing Guo

**Affiliations:** a Institute of Pharmaceutical Science, King’s College London, London, UK; b Biomanufacturing Research Centre, School of Mechanical and Electrical Engineering, Soochow University, Su-Zhou, China

**Keywords:** Nano spray-drying, pulmonary protein delivery, alkaline phosphatase, aerodynamic particle size distribution, derived metrics, USP Chapters <601> and <1604>

## Abstract

Pulmonary protein delivery has proven to be an effective approach for the treatment of local and systemic diseases, and particles that can be efficiently inhaled into peripheral regions should possess aerodynamic sizes of <2 µm, for which nano-spray-drying (NSD) is a powerful tool for fabrication. However, NSD powders have a high specific surface area and surface energy, which causes severe agglomeration and poor aerosolization performance. To solve these problems, cyclodextrin/leucine delivery systems were constructed, which can evidently protect alkaline phosphatases (AKP) with >94% of their biological activities retained in NSD powders and endow nanoparticle-based AKP-cyclodextrin-leucine (ACL) powders with improved aerosolization behavior. This can be evidenced by the fine particle (<4.46 µm) fraction (FPF_<4.46_) and FPF_<1.66_ (in unit percentage), of only 49.9% and 18.7% for the leucine-free ACL0, while significantly increased to 82.1% and 33.1% for ACL15, and further increased to >88% and >41% for ACL30/ACL45. The USP Chapters of traditional <601> and newly published <1604> were employed to determine derived metrics for aerodynamic particle size distribution (APSD). It was demonstrated that the authenticity of fitted values calculated in Chapter <601> was severely influenced by APSD profiles, and the fitted values can have immense differences from the actual values determined in Chapter <1604> . The differences can be minimized when the APSD is unimodal and normal across sized impactor stages. Hence, the authenticity of the fitted values can be improved. These new data deepen the understanding of APSD analyses and generate insights into the improvement of value authenticity for derived metrics, henceforward facilitating continuous improvements in quality control of inhaled products.

## Article highlights

- Cyclodextrin effectively protects thermolabile proteins during the nano spray-drying process.
- Leucine can successfully enhance dispersibility and aerosolization manners for nanoparticles.
- Authenticity of fitted values for derived metrics in the Chapter is determined by APSD profiles.
- The values for a derived metric can be very different as determined by <601> and <1604>.
- Unimodal/normal APSDs minimize differences between fitted and actual values for derived metrics.

## Graphical abstract

**Figure:**
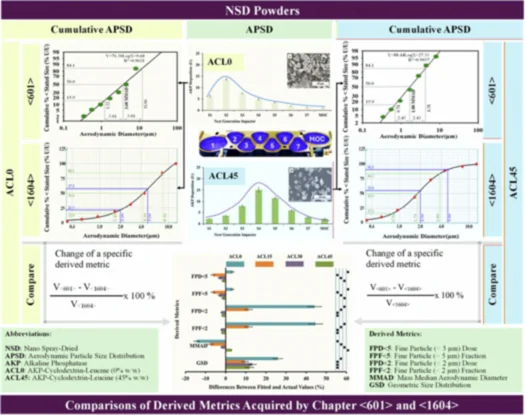

## 1. Introduction

Dry powders are the primary formulation for pulmonary delivery of biologics, as they offer distinctive advantages such as improved stability, high dose, large design space, and avoidance of cold chain for storage and transportation. Pulmonary delivery of proteins can be utilized for the treatment of local (e.g. asthma, COPD, lung cancer, etc.) and systemic diseases (e.g. diabetes, growth disorder, and infertility) (Chang et al. 2021). Spray drying has proven to be a powerful tool for fabricating inhalable dry powders in one step and offers an ample space for designing particles with the desired size, shape, density, surface morphology, and surface energy (Li et al. 2003; Li et al. 2005a, 2005b; Li and Birchall 2006, Seville et al. 2007, 2010; Li and Seville 2010; Yang et al. 2015a, 2015b; Alhajj et al. 2021; Li and Xu 2023). In addition, for drug delivery to the lungs, there are strict requirements on the aerodynamic sizes of dry powders; only those with a size of < 5 µm can be inhaled into the lower respiratory tract, and those with sizes of < 2  µm can be further conveyed to alveolar regions that are the major site targeted for pulmonary delivery (Carvalho et al. 2011). For the preparation of dry powders with a size of < 2 µm, the recently developed nano-spray-drying (NSD) is a powerful tool for achieving this purpose. NSD utilizes the high-frequency mesh vibration to turn a bulk drug solution into numerous micronized droplets (3–8 µm), inside which the solvents can then be evaporated swiftly in a heated chamber to quickly produce dry particles with a size largely at nanosized scales and as miniature as 0.3 µm (Arpagaus 2018). NSD has demonstrated great potential for the preparation of nanoparticle-based systems for drug delivery to deep lungs via inhalation (Jadhav et al. 2025). However, in comparison with mini spray-dried powders, NSD particles have massively enlarged specific surface areas and increased surface energy, which subsequently cause them to have much augmented cohesiveness and a greater tendency to aggregation (Schmid et al. 2011), leading to reduced powder dispersibility and poor aerosolization behavior. In our previous studies, amino acids, particularly leucine, were shown to reduce surface energy, thus capably enhance the dispersibility and aerosolization performance for spray-dried powders (Li et al. 2003, Li et al. 2005a; Yang et al. 2015a), and cyclodextrin was exhibited to effectively increase the surface roughness of spray-dried particles, henceforth diminishing interparticle contacts, leading to the improvement of powder dispersibility and aerosolization behavior (Li et al. 2005b). Since then, these findings have strongly supported other following studies on inhalable spray-dried powders and are further confirmed by their data (Cabral-Marques and Almeida 2009; Dufour et al. 2015; Wang et al. 2021; Zhang et al. 2024). However, these investigations were conducted on mini-spray-dried powders, and no or very limited data disclosed how these excipients can affect the delivery of NSD powders to the lungs (Kaewjan and Srichana 2016). Despite a recent study reporting the use of cyclodextrin derivatives as excipients in NSD powders, their role was to form inclusion complexes to improve the water solubility and bioavailability of a hydrophobic drug. For achieving such purposes, extremely water-soluble cyclodextrin derivatives were utilized, including sulfobutylether-*β*-cyclodextrin and (2-hydroxy-3-*N*,*N*,*N*-trimethylamino) propyl-*β*-cyclodextrin chloride, which can allow the insertion of poorly water-soluble drug molecules into their cavities to form complexes, thereby greatly improving the drug solubility in water. However, these highly water-soluble cyclodextrin derivatives distinctively augmented the water content and hygroscopicity of NSD powders, thereby causing poor aerosolization performance and low lung deposition (Motzwickler-Németh et al. 2025). Therefore, their report had a dissimilar primary research purpose and consequently was not comparable to our study.

Additionally, for the preparation of spray-dried powders of biologics, it is necessary to employ an appropriate thermo-protectant in the formulation to shield biomacromolecules from heat during spray-drying and to support their three-dimensional (3D) chemical structures, thus maintaining their biological functions in the dry state. Our previous studies have shown that disaccharides (i.e. lactose and trehalose) (Li et al. 2003, 2005a, 2005b) and polysaccharides (i.e. chitosan and cellulose) (Li and Birchall 2006; Li et al. 2010; Li and Seville 2010) can effectively preserve the biological functions of biomacromolecules (i.e. genes and proteins) during the spray-drying process. However, oligosaccharides such as cyclodextrin have not been well investigated for the protection of biologics, particularly in the NSD process, where droplets are much smaller in size (3–8 µm) (Arpagaus 2018) than those (60–80 µm) generated by a mini spray dryer (Yu et al. 2016). Hence, the heat transfer throughout the body of NSD droplets would be greatly increased, which consequently affects the 3D structures and biological functions of biologics.

Alkaline phosphatase (AKP) is an enzyme widely distributed in the human body and plays a pivotal role in biological processes such as bone mineralization and transportation of molecules in the intestines. AKP can catalyze the removal of phosphates from a variety of molecules, and in vitro, AKP can uniquely catalyze the hydrolysis of para-nitrophenyl phosphate (PNPP) to produce para-nitrophenol (PNP), which is yellowish and soluble in alkaline aqueous solutions and hence can be quantified colorimetrically. Based on this working rationale, the activities of AKP can be calculated by the amount of PNP produced within a defined time interval during the catalytic reaction (Breslow and Katz 1968; Tang et al. 2019). Additionally, AKP is dimeric and contains two identical monomers. Each monomer has 429 amino acids and three metal ions (2 x Zn^2+^ and 1x Mg^2+^) that are essential for its catalytic activity, and the two monomers are connected by disulfide bonds to form an AKP biomacromolecule with a complex 3D structure (Coleman 1992; Vimalraj 2020). This subtle and sophisticated structure makes AKP biomacromolecules very sensitive to heat, and they are labile and intended to be denatured in heat, thereafter losing their biological functions (Dumitrașcu et al. 2015). This is supported by our previous data where the unprotected AKPs largely lost their activities in the spray-drying process, with only ~70% w/w of biological activities retained in spray-dried powders (Li et al. 2010).

Aerodynamic particle size distribution (APSD) is a critical quality attribute for orally inhaled products (OIPs) (FDA 2018), and the evaluation of APSD for an OIP uses a cascade impactor, typically of the Next Generation Impactor (NGI) that operates on the rationale of inertial impaction to fractionate aerosolized particles or droplets according to their sizes in aerodynamic diameter (D_ae_). Based on APSD data, the cumulative mass percentages less than the stated sizes were then calculated and plotted against the logarithm of aerodynamic sizes in diameter to construct the cumulative APSD, and the derived metrics were subsequently determined, including the fine particle fraction (FPF), mass median aerodynamic diameter (MMAD), and geometric standard deviation (GSD), which are key parameters for evaluating the pulmonary deposition efficiency of inhalable formulations. For cumulative APSDs, two Chapters from the United States Pharmacopeia (USP) define the calculations and constructions. The normative Chapter <601> is a traditional regulation and is still widely accepted in the industry sector, while the informative Chapter <1604> has recently been published and reveals important differences in methodologies for the analyses of APSD. In Chapter <601> , the mass impacted on all stages is summated as the total mass for the calculation of cumulative mass percentages less than the stated sizes that are subsequently plotted in probability against the aerodynamic sizes in logarithm to produce a linear regression equation as the theoretical model for cumulative APSD, and this linear model is employed for the calculations of fitted values for derived metrics (USP <601> 2023a). In Chapter <1604> , only the mass impacted on the sized impactor stages counts for the calculations of cumulative mass percentages less than the stated sizes that are plotted on a normal scale against aerodynamic sizes in logarithm, between which a best-fit curve is constructed as the cumulative APSD that is utilized for the evaluation of actual values for derived metrics. Additionally, Chapter <1604> also regulates the stage groupings, categorized as non-sized particles, coarse particles, fine particles, and extra-fine particles, which individually refer to those particles that do not have size limits and are impacted in upper airways, lower airways, and alveolar regions (USP <1604> 2023b). At a flow rate of 60 L/min for the NGI with a pre-separator, non-sized particles refer to those collected from the induction port and pre-separator, coarse particles from stages 1 and 2 (D_ae_: 12.8–4.46 µm), fine particles from stages 3, 4 and 5 (D_ae_: 4.46–0.94 µm), and extra fine particles from stages 6, 7 and MOC (D_ae_: 0.94–0.14 µm) (Roberts and Mitchell 2019). Apart from these differences, large deviations can also be generated between the fitted values and actual values for a specific derived metric individually determined from cumulative APSDs constructed following Chapters <601> and <1604> , and the magnitude of deviation is related to APSD profiles across sized impactor stages. To date, no reports have revealed the implementation of these two Chapters into a real situation to disclose the differences between fitted values calculated in Chapter <601> and actual values evaluated in Chapter <1604> , and further to look into the relationships between APSD profiles and the authenticity of fitted values.

Therefore, this study was aimed to fabricate NSD protein powders with well-preserved biological functions and exceptional dispersibility for efficient delivery to deep lungs, and further to analyze APSD by implementing traditional Chapter <601> and recently published Chapter <1604> for the generation of insights into intrinsic relationships among APSD profiles, fitted values, and actual values of derived metrics, henceforward to improve data accuracy for precisely characterizing APSD. The main objectives consist of two aspects. The first is to increase the fine particle (<1.66 µm) fraction (FPF_<1.66_) to over 40% for NSD powders by a cyclodextrin/leucine composite system; and the second is to reveal the influence of APSD characteristics (i.e. unimodal, normal, and abnormal) on the differences between fitted values and actual values for derived metrics, hence, to provide a basis for improving the quality control of inhaled products. For achieving these purposes, cyclodextrin is employed as an excipient to protect the biological function of heat-sensitive AKP (model protein) and to endow NSD powders with satisfactory dispersibility. Leucine was employed and leveled to enhance the dispersibility of the nanoparticle-based NSD powders. Furthermore, NGI with a pre-separator was utilized to evaluate the APSDs for the NSD powders, so that all impactor stages were sized to produce the same set of data of cumulative mass percentages less than the stated sizes versus cut-off sizes in aerodynamic diameter. These data were then plotted to produce cumulative APSDs following Chapters <601> and <1604> , from which the derived metrics were individually determined and compared to generate insights into the relationships between the APSD profiles, fitted values, and actual values for further improvement in quality control of inhaled products.

## 2. Materials and methods

### 2.1. Materials

AKP from the bovine intestinal mucosa (lyophilized powder, >10 U/mg; catalogue number (CN): P7640), *p*-nitrophenyl phosphate disodium salt (CN: 4876), and *p*-nitrophenol (CN: 1048) were purchased from Sigma-Aldrich (St. Louis, MO, USA). Diethanolamine (CN: 40014160), magnesium chloride (CN: TM368825G), *β*-cyclodextrin (CN: 69009360), leucine (CN: XW33105816), and HCl (37% w/w; CN: FH1200PB1701) were acquired from Sinopharm Chemical Reagent Co., Ltd. (Shanghai, China). All reagents were of analytical grade and were used as received.

### 2.2. Preparation of NSD powders

Four spray-drying solutions were prepared according to the experimental design as listed in Table 1. For preparation, AKP, *β*-cyclodextrin, and leucine in defined amounts were fully dissolved in distilled deionized water (ddH_2_O) to produce working solutions. For spray-drying, a laboratory-scale nano spray dryer (Büchi Nano Spray Dryer B-90, Büchi Labortechnik AG, Switzerland) was used to prepare NSD powders. The standard operation parameters were as follows: inlet temperature, 105 °C; air flow rate, 130 L/min; spray rate, 100%; spray cap with mesh at a pore size of 4.0 µm; and outlet temperature adjusted to 53 °C. The NSD powder was captured via electrostatic adsorption, collected in a glass vial, and stored in a vacuum desiccator for further use. This process was repeated six times for each formulation.

**Table 1.** The preparation of spray-drying solutions and characterization of physicochemical properties for NSD powders.

Samples	Spray-drying solutions					NSD powders			
	AKP (mg)	Excipients			Water (mL)	Yield (%, w/w)	Moisture content (%, w/w)	Retained specific activity of AKP	
		*β*-cyclodextrin (mg)	Leucine					(U/mg)	(%, U/U)^†^
			(mg)	(%, w/w)					
ACL0	200	1200	0	0	100	47.9(4.6)^I^	6.4(0.3)^I^	12.42(0.45)^I^	93.9(1.6)^I^
ACL15	200	1020	180	15	100	51.8(2.1)^I^	5.6(0.3)^II^	12.64(0.38)^I^	95.5(2.2)^I^
ACL30	200	840	360	30	100	48.6(3.1)^I^	4.5(0.2)^III^	12.89(0.64)^I^	97.4(2.5)^I^
ACL45	200	660	540	45	100	51.4(3.8)^I^	3.7(0.2)^IV^	12.70(0.59)^I^	96.0(1.8)^I^

For NSD powders, the physicochemical properties were compared, and the different Roman numbers (i.e. I, II, III, and IV) in the same column represented significant differences among values. The data are expressed as mean ± S.D., *n* = 3.

^†^ The percentage of retained AKP activity was calculated by dividing the retained AKP-specific activity in the NSD powders by that (13.32 U/mg) of the raw AKP.

### 2.3. Visualization of surface morphology

The surface morphology, shape, and approximate size of the NSD particles were visualized by scanning electron microscopy (SEM, S-4700, Hitachi, Tokyo, Japan), operated at 15 kV under high vacuum. For the generation of electrical conductivity on the surface, samples of NSD particles were sputter-coated with a thin layer of gold for 90 s under vacuum (HITACHI E-1010, Tokyo, Japan). Typical micrographs of the NSD particles were captured and shown.

### 2.4. Particle size distribution (PSD)

The PSD of the NSD powders was determined by laser diffraction (Mastersizer 2000E, Malvern, UK) using a dry powder dispenser (SCIROCCO 2000). Approximately 200 mg of NSD powder was weighed to accomplish the required obscuration of 0.5%–5%. Nitrogen flow (4 bar) was used to aerosolize and carry dry powders through the measurement cell, and the PSD was subsequently determined according to the laser diffraction pattern. Each sample was measured in triplicate. The PSD is expressed in terms of the Span factor, which is calculated as Span = (*d*[*v*, 90]—*d*[*v*, 10])/*d*[*v*, 50], where *d*[*v*, 10], *d*[*v*, 50], and *d*[*v*, 90] are volume-weighted particle sizes with an agreed percentage of particles smaller than the stated size. A smaller Span value indicates a narrower particle size distribution.

### 2.5. Moisture content

The moisture contents of the NSD powders were determined by thermogravimetric analysis (TGA 500, TA Instruments, New Castle, USA). The samples (approximately 5 mg) were placed in platinum pans and analyzed under a nitrogen purge (20 mL/min) over the temperature range of 20–140 °C at a heating rate of 10 °C/min. Measurements were performed in triplicate.

### 2.6. AKP activity assay

The AKP contents in the NSD powders were determined using a quantitative assay as described in our previous studies (Neumann and Van Vreedendaal 1967; Li et al. 2010). Briefly, a known amount of powder (control = raw AKP) was dissolved in diethanolamine–HCl (1 M, pH 9.8, 0.5  mM MgCl_2_) buffer to prepare a sample solution of an appropriate concentration for the quantitative assay. A 10 µL aliquot of the sample solution was subsequently added to 2.49 mL of freshly prepared and warmed (37 °C) substrate solution (16 mM PNPP in diethanolamine–HCl buffer) and incubated at 37 °C for 60 s, during which PNPP was converted to PNP through the biochemical reaction catalyzed by AKP. The absorbance of the solution was determined by UV–vis spectrometry at 405 nm. The final concentration of PNP was then calculated by comparing the absorbance against the PNP calibration curve constructed using a series of standard solutions of PNP (0–16 µM) in diethanolamine-HCl buffer, which were in the verified range of PNP concentrations (0–100 µM) for producing a linear regression equation (Liu et al. 2025). One unit (U) of AKP was defined as the amount of AKP required for catalyzing the substrate to produce 1 µmol PNP within 1 min at 37 °C. Hence, the amount of AKP in units was subsequently determined for a sample, to represent the biologically active mass of AKP deposited in that stage.

### 2.7. In vitro aerosolization performance

The in vitro aerosolization performance of NSD powders from a dry powder inhaler (DPI) device was determined using an NGI (Copley Scientific, Nottingham, UK) equipped with a USP throat and a pre-separator. NGI is an eight-stage inertial impactor that fractionates aerosolized powders into isolated groups according to their aerodynamic sizes. For the test, the NSD powder (~20 mg, equivalent to 35 U of AKP) was accurately weighed, loaded into a gelatin capsule (size 3), and then placed into a Cyclohaler® device that was subsequently connected to the USP throat via a mouthpiece adapter. The powders were aerosolized (35% relative humidity, 20 °C) at 60 L/min for 4 s, and two capsules (~40 mg, equivalent to 70 U of AKP) were aerosolized for each test. After deposition, the capsule and inhaler device, throat, pre-separator, stages 1–7, and micro-orifice collector (MOC) were individually rinsed with 10 mL of diethanolamine–HCl buffer for the collection of impacted powders, and AKP was then quantified via an activity assay. For each test, the total dose (TD) was defined as the total amount of AKP loaded into the capsules (theoretically 70 U). The recovered dose (RD) was defined as the cumulative amount of AKP collected from all parts, including the capsules, inhaler, throat, pre-separator, stages 1–7, and MOC, and its expression as a percentage of TD was named the recovered fraction (RF). The emitted dose (ED) was defined as the amount of AKP emitted from the device (i.e. throat, pre-separator, stages 1–7, and MOC) during aerosolization, and the expression as a percentage of RD was named the emitted fraction (EF). The inhalation dose (ID) was defined as the units of AKP deposited in stages 1–7 and the MOC, and the expression as a percentage of RD was defined as the inhalation fraction (IF). The fine particle dose (FPD), termed as FPD_<4.46_ and FPD_<1.66_, was defined as the cumulative mass of AKP collected from stage 3 to MOC (effective cut-off diameter, ECD < 4.46 μm) and from stage 5 to MOC (ECD < 1.66 μm) of the NGI, respectively. The FPF_<4.46_ and FPF_<1.66_ were determined as the mass percentage of FPD_<4.46_ and FPD_<1.66_ to ID, respectively (Li and Xu 2023; USP <601> 2023a).

### 2.8. Derived metrics calculated from USP Chapter <601>

To further characterize the APSD, the derived metrics were subsequently determined, including FPD_<5_ (D_ae_ < 5 μm), FPF_<5_, FPD_<2_ (D_ae_ < 2 μm), FPF_<2_, MMAD, and GSD. According to Chapter <601> , the mass of AKP impacted on stages 1–7 and MOC were summed, against which the mass percentage of AKP deposited on each stage was calculated accordingly. The cumulative APSD was constructed by plotting the cumulative percentages of AKP less than the stated size in aerodynamic diameter in probability scale on the ordinate against the logarithm of cut-off aerodynamic size in diameter on the abscissa. A linear regression equation was then generated as the model in statistics for cumulative APSD (*p* < 0.05), and the coefficient of determination (R^2^) signified the level of goodness-of-fit between the theoretical model and experimental data. Using this linear equation, the derived metrics of FPD_<5_, FPF_<5_, FPD_<2_, and FPF_<2_ were subsequently calculated, and further, the MMAD and GSD were determined for a complete description of the APSD. MMAD, described as a mass-weighted median size, refers to the specific value on the abscissa where the regressed line crosses the other line that passes through the 50-percentile point on the ordinate and is parallel to the abscissa. The GSD designates the breadth of particle sizes in aerodynamic diameter, and a smaller value of the GSD suggests a narrower distribution of aerodynamic sizes for particles in an aerosol. The value for the GSD can be determined according to the formula of GSD = [D_84.1_/D_15.9_]^1/2^, in which D_84.1_ and D_15.9_ are the aerodynamic sizes in diameters corresponding to the cumulative percentages of mass at 84.1% w/w and 15.9% w/w, respectively (Li et al. 2025; USP <601> 2023a).

### 2.9. Derived metrics evaluated from USP chapter *<1604>*

The derived metrics evaluated from the USP Chapter <1604> were entirely different. According to Chapter <1604> , aerosolized powders must be grouped according to the sites where they are impacted. At 60 L/min and with pre-separator, the non-sized particle (D_ae_ > 12.8 µm) dose (NPD_<1604>_) refers to the cumulative amount of AKP deposited in throat and pre-separator (Group 1); The coarse particle (12.8 µm > D_ae_ > 4.46 µm) dose (CPD_<1604>_) refers to the sum of AKP impacted into stages 1 and 2 (Group 2); The fine particle (4.46 µm > D_ae_ > 0.94 µm) dose (FPD_<1604>_) is the total amount of AKP collected from stages 3, 4 and 5 (Group 3); And the extra-fine particle (0.94 µm > D_ae_ > 0.14 µm) dose (EPD_<1604>_) represents the entirety of AKP impacted into stages 6, 7, and MOC (Group 4). The expression as a percentage of ED for NPD_<1604>_, CPD_<1604>_, FPD_<1604>_, and EPD_<1604>_ was sequentially defined as non-sized particle fraction (NPF_<1604>_), coarse particle fraction (CPF_<1604>_), fine particle fraction (FPF_<1604>_), and extra-fine particle fraction (EPF_<1604>_). Additionally, for the evaluation of all other derived metrics (revealed in Section 2.8) by following Chapter <1604>, the cumulative mass percentages of AKP less than the stated cut-off aerodynamic diameters were plotted on a normal scale on the ordinate against the logarithm of aerodynamic sizes on the abscissa to produce a best-fit curve. According to this best-fit curve, the values were evaluated for the derived metrics of FPD_<5,<1604>_, FPF_<5,<1604>_, FPD_<2,<1604>_, FPF_<2,<1604>_, MMAD_<1604>_, and GSD_<1604>_ (Roberts and Mitchell 2019; USP <1604> 2023b).

### 2.10. Differences of values for derived metrics

Each of these critical derived metrics of FPD_<5_, FPF_<5,_ FPD_<2_, FPF_<2_, MMAD, and GSD had a fitted value of V_<601>_ and actual value of V_<1604>_ determined by Chapters <601> and <1604> , respectively. The difference in percentage between these two values for a specific derived metric was subsequently calculated according to the equation of (V_<601>_−V_<1604>_) ∗ 100/V_<1604>_ to reflect the level of authenticity of the fitted value, and a minor difference suggests a higher degree of validity for the fitted value.

### 2.11. Statistics analysis

All data are expressed as the mean ± standard deviation (S.D.), and the values for both parameters are acquired by using Excel. Statistical analysis was performed using one-way ANOVA (IBM SPSS Statistics for Windows, Version 29.0.2.0, Released 2023, IBM Corp. Armonk, NY, USA), and Duncan’s test was employed as a post-hoc analysis to determine the difference, and *p* < 0.05 was statistically significant. The ‘significance’, ‘significant’, or ‘significantly’ mentioned in this report generally referred to the parameters that were statistically analysed by ‘one-way ANOVA, Duncan’s test, *p* < 0.05′ unless otherwise stated. The sample size (*n*) was 3, which was based on the coefficient of variation from preliminary experiments.

## 3. Results

### 3.1. Quantitative assay of AKP

AKP activity was determined by colorimetrically quantifying the product of PNP in the catalytic reaction of enzymatically degrading PNPP, and one unit of AKP was defined as the amount of AKP required to generate 1 μmol of PNP per minute at 37 °C. A calibration curve was constructed by plotting the absorbance at 405 nm versus a series of PNP standard concentrations (0–16 μM), and a linear relationship was obtained using the equation of Y = 0.0186X + 0.0044 (R^2^ = 0.9997), which can produce the recovery of 99.9 ± 0.3% w/w (data not shown) and was subsequently employed for the quantification of AKP activities. As NSD powders were comprised of a model protein of AKP and excipients of *β*-cyclodextrin/leucine that occupied mass fractions of 0%, 15%, 30%, and 45% w/w, respectively, they were subsequently named as ACL0, ACL15, ACL30, and ACL45, respectively, in sequence. It was revealed that all NSD formulations can well preserve biological functions for AKP, and the specific activity (the total activities per theoretical milligram of AKP) was in the range of 12.42–12.89 U/mg, representing 93.9%–97.4% U/U of enzymatic activities still retained in NSD powders as compared to that (13.23 U/mg) of raw AKP; And there were no significant differences observed among these values (Table 1).

### 3.2. General physical characteristics of NSD powders

Under the operating conditions for spray-drying these four formulations, the NSD yields were individually 47.9 ± 4.6% w/w for ACL0, 52.8 ± 2.1% w/w for ACL15, 48.6 ± 3.1% w/w for ACL30, and 51.4 ± 3.8% w/w for ACL45, with no significant differences identified among these values (Table 1).

The residual moisture contents of the NSD powders were investigated by thermogravimetric analysis and determined by the mass loss of powders at temperatures of 20–140 °C (Table 1). It was demonstrated that the moisture content was 6.4% w/w for ACL0, then significantly reduced to 5.6% w/w for ACL15, further diminished to 4.5% w/w for ACL30, and finally decreased to 3.7% w/w for ACL45. Clearly, the increase of leucine in the formulation can reduce the moisture content.

**Figure 1.**
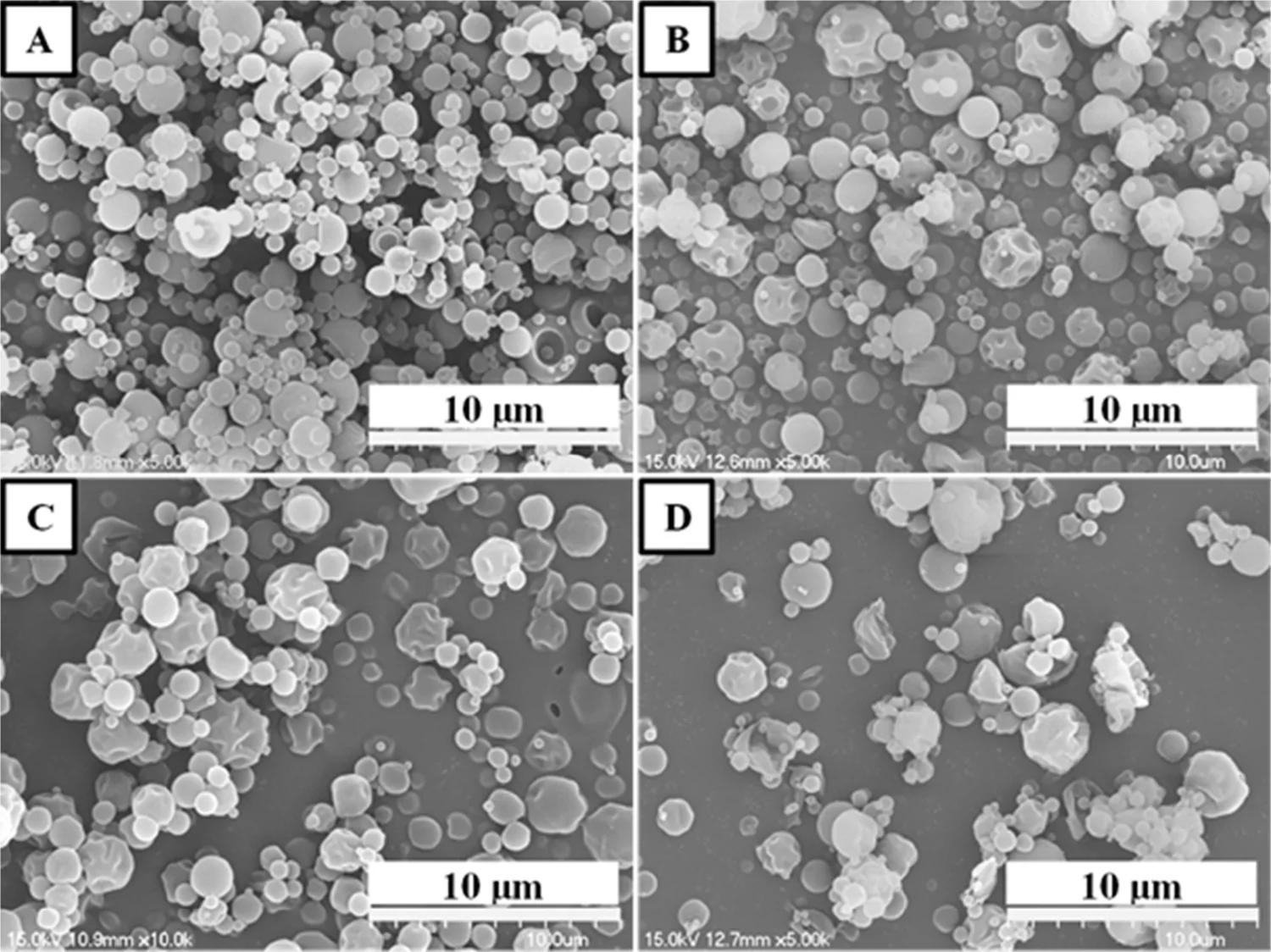
The size, shape, and surface morphology of NSD powders visualized by SEM. (A) ACL0; (B) ACL15; (C) ACL30; (D) ACL45. Bar = 10 µm.

SEM was used to observe the particle size, shape, and surface morphology of the NSD powders (Figure 1). In terms of size, all NSD powders were primarily less than 2 µm and mainly in the nanosized range (0.3–1 µm), which were entirely agreed with the data reported by the manufacturer of nano spray-dryer (Li et al. 2010). With respect to their shape and morphology, the ACL0 particles were fundamentally spherical with a smooth surface, except for a small number of particles with a sunken area (‘cavity’) on the surface (Figure 1A). The ACL15 particles predominately displayed golf-ball shapes (Figure 1B), to which the majority of ACL30 had comparable silhouettes, and some particles had a collapsed surface (Figure 1C). This tendency was strengthened with the addition of extra leucine, and an increasing number of ACL45 particles were observed with collapsed surfaces and irregular shapes (Figure 1D). Clearly, the presence of leucine in the formulations disrupted the surface of NSD powders, hence increased their surface roughness, which was entirely in agreement with previous data in which the addition of leucine into formulations at high concentrations introduced collapsed surfaces to spray-dried particles (Mangal et al. 2015). Additionally, the distances between NSD particles were extended on average as the leucine content in the formulations increased, suggesting diminished interparticle cohesiveness, and further supporting enhanced powder dispersibility and improved aerosolization behavior. These observations were entirely agreed with our previously reported data (Li et al. 2003).

**Figure 2.**
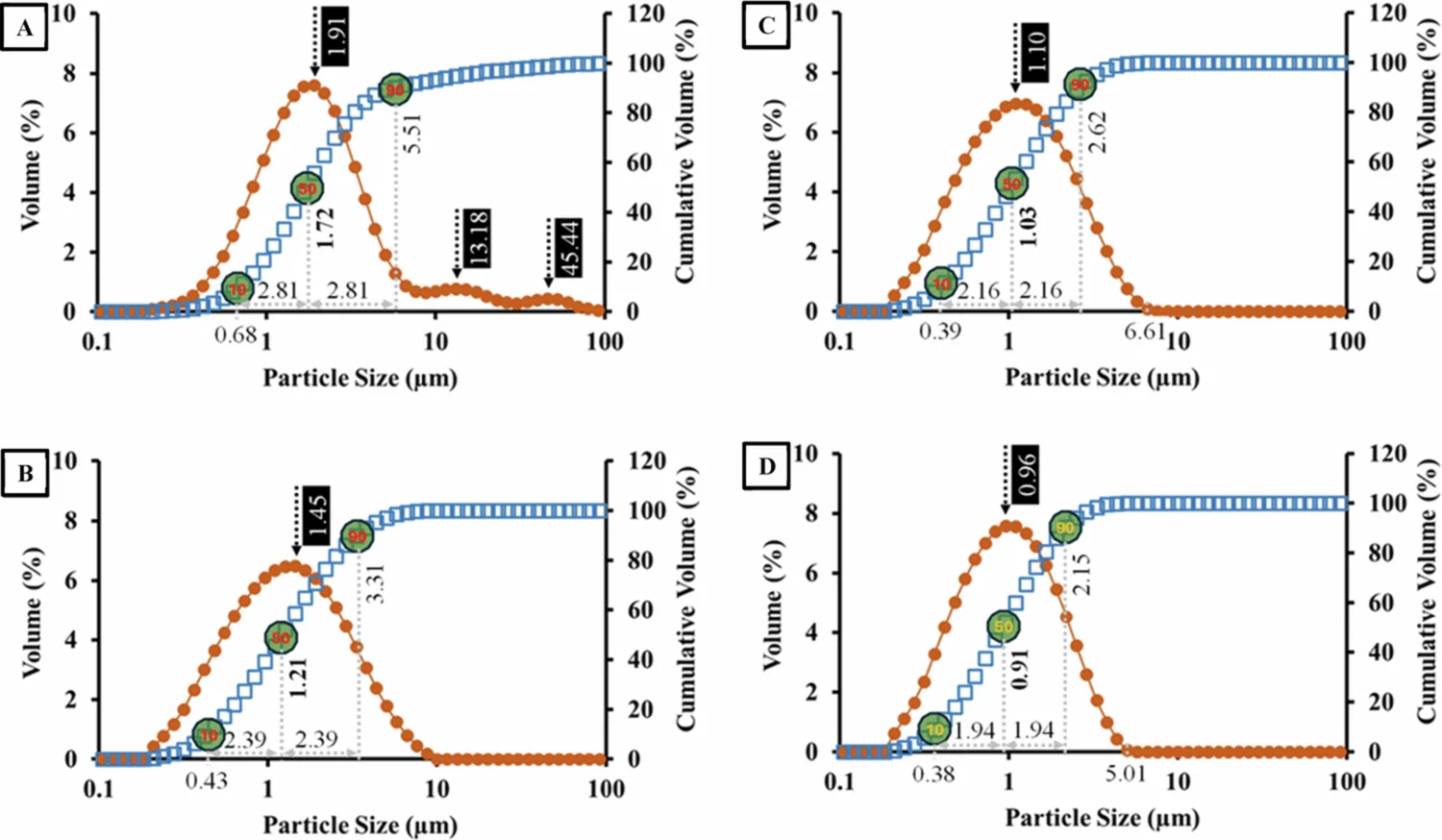
Particle size distribution of NSD powders determined by laser diffraction. (A) ACL0; (B) ACL15; (C) ACL30; (D) ACL45. The green solid circles with the numbers 10, 50, and 90 represented that the cumulative percentages of powders in the volume were 10%, 50%, and 90%, respectively, at which, the *d*[10, *ν*], *d*[50, *ν*], and *d*[90, *ν*] were specified. The numbers in black rectangles were the modes of the particle size distributions. The Span was then calculated and shown on the horizontal arrow for each NSD powder, suggesting the width of the particle size distribution.

To further evaluate the physical size of NSD powders, laser diffraction was employed to determine the PSD (Figure 2). The PSD of each powder was diagrammed and analyzed to provide insights into particle interactions and their actual situations in airflow. The PSD for ACL0 powders was multimodal (Figure 2A), with a major (93.3% in volume) size < 10 μm in diameter (named as ACL0-PSD1 that was followed for sub-PSD labeling), a second population (4.4% in volume) with a particle size < 30 μm in diameter (ACL0-PSD2), and a third population (2.3% in volume) < 100 μm (ACL0-PSD3). Considering that the ACL0 particles were predominately < 2 μm and many of them were in the nanosized range (Figure 1A), these data indicated that there was strong interparticle cohesiveness in the ACL0 powders, which caused the formation of aggregates that failed to be broken up by the instrument air stream for dispersion, as illustrated in previous reports (Li et al. 2003; Li and Xu 2023; Li et al. 2005a, 2005b). In comparison with the mode (1.91 μm) for ACL0-PSD1, the modes for ACL0-PSD2 and ACL0-PSD3 were distinctively increased to 13.18  μm and 45.44 μm, suggesting that the aggregates formed from approximately 7 and 24 single NSD particles, respectively. For the overall PSD of ACL0 powders, the *d*[*v*, 10], *d*[*v*, 50], and *d*[*v*, 90] were sequentially of 0.68, 1.72, and 5.51 µm, respectively, with a Span of 2.81. In contrast, the addition of leucine in formulations can gradually eliminate aggregation, thereby progressively enhancing the dispersibility of NSD powders. It was demonstrated that the ACL15 powder displayed a unimodal PSD of < 10 µm with a mode of 1.45 µm, and the *d*[*v*, 10], *d*[*v*, 50], and *d*[*v*, 90] were of 0.43, 1.21, and 3.31 µm, respectively, resulting in a Span of 2.39 (Figure 2B). The ACL30 powder also demonstrated a unimodal PSD while of < 6.61 µm with a mode of only 1.10 µm, and the *d*[*v*, 10], *d*[*v*, 50], and *d*[*v*, 90] were reduced to 0.39, 1.03, and 2.62 µm, respectively, leading to a shrunken Span of 2.16 (Figure 2C). Unsurprisingly, the ACL45 powders exhibited a unimodal PSD where the maximum size was further reduced to 5.01 µm with the mode lowered to 0.96 µm, and the *d*[*v*, 10], *d*[*v*, 50], and *d*[*v*, 90] were expectedly diminished to 0.38, 0.91, and 2.15 µm, respectively, resulting in a further decreased Span of only 1.84 (Figure 2D). Obviously, the continuous increase in leucine content in the formulation can incessantly decrease the mode, *d* [*v*, X], and Span for NSD powders, strongly suggesting that powder dispersibility is constantly enhanced, further indicating that interparticle cohesiveness is consecutively lessened.

### 3.3. In vitro aerosolization performance

**Figure 3.**
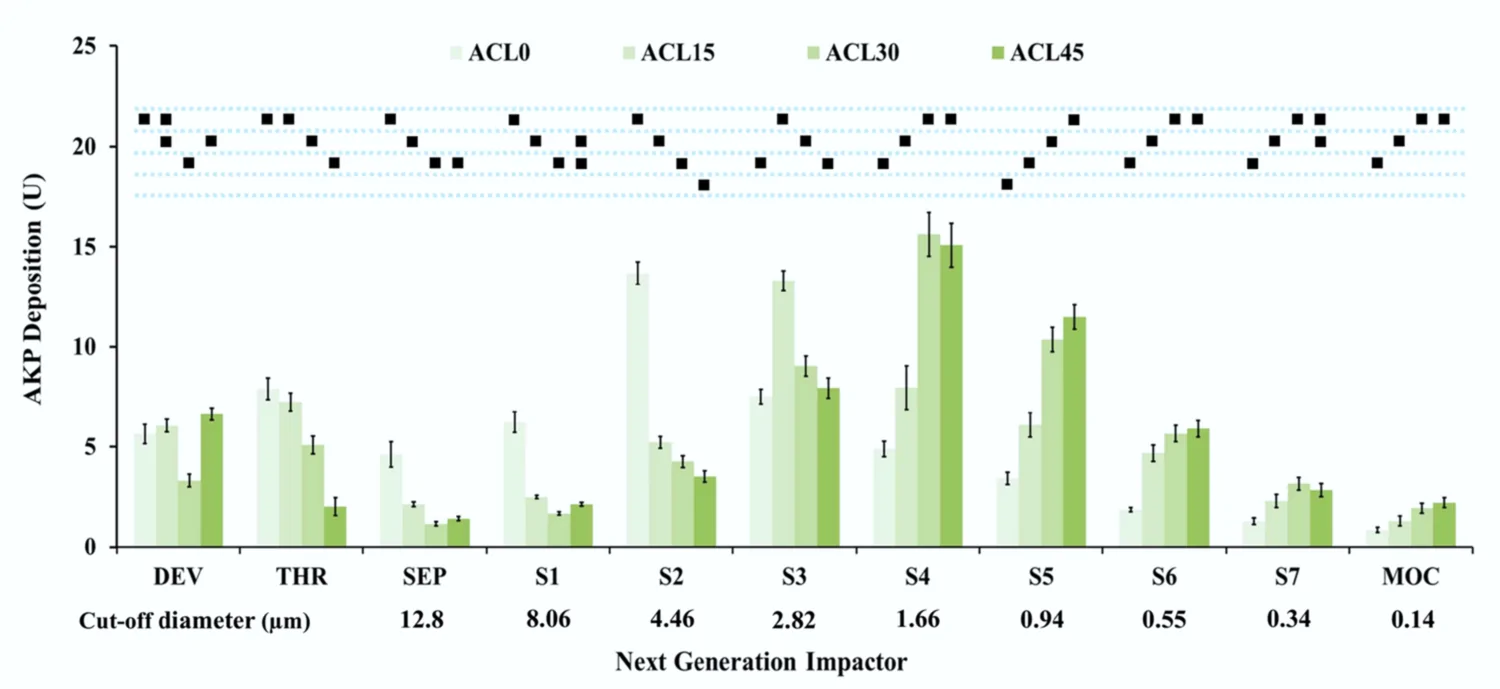
In vitro deposition of NSD powders was determined by NGI. A certain amount (~20 mg, equivalent to ~35 U of AKP) of NSD powder was accurately weighed into a gelatine capsule (size 3), then placed into Aerolizer® , which was subsequently connected to the NGI through an adapter. The NSD powders were aerosolized (35% of relative humidity, 20 °C) at 60 L/min over 4  s. For each powder, two capsules (40 mg/70 U) were prepared and aerosolized to evaluate the APSD. After deposition, the powders impacted on each NGI stage were individually collected by the buffer solution (10 mL), and the AKP was quantified (in units) colorimetrically via the catalytic reaction on its substrate. Each powder was tested in triplicate. The deposition data at each part for all NSD powders were statistically compared by one-way ANOVA (Duncan’s test, *p* < 0.05), and the sign of black solid squares on different horizontal lines represented significant differences. Data were expressed as mean ± S.D., *n* = 3.

NGI was used to evaluate the in vitro aerosolization performance of NSD powders. For the evaluation, the TD was scheduled to be 70 U (two capsules) for each NSD powder, and the results of APSD data are shown in Figure 3 and listed in Table 2. Clearly, all NSD powders showed RD of 57.9–61.3 U and RFs of 82.7%–87.6% U/U, with no significant difference detected. The ED was the total AKP amount collected from the throat, pre-separator and all stages of impactor, suggesting the flowability of NSD powders from capsules. The EF was the mass percentage calculated through dividing ED by RD. The ED was 52.3 U for ACL0 and then increased to 52.7–58.0 U for ACL15, ACL30, and ACL45, suggesting that leucine-modified NSD powders have improved powder flowability, and this assertion can be further supported by EF, which was 90.2% U/U for ACL0 while significantly increased to 94.6% U/U for the leucine-modified NSD powders of ACL30. The ID is the cumulative amount of AKP collected from stages 1 to 7 and the MOC, and the IF is the fraction of ID over RD. The IDs and IFs suggest the dispersibility of NSD powders in airflow and further indicate the powder delivery to the pharynx, larynx and lower respiratory tract. Clearly, the ID and IF were individually 39.8 U and 68.7% U/U, respectively, for ACL0, then significantly increased to 43.4 U and 73.7% U/U, respectively, for ACL15, and further augmented to ~51 U and ~84% U/U, respectively, for ACL30 and ACL45. These data strongly support that the presence of leucine in formulations can distinctively enhance the dispersibility of nanoparticle-based NSD powders and that this tendency can be intensified with an increase in leucine content. The FPD_<4.46_ and FPF_<4.46_ represented the amount and fraction of NSD powders that can be inhaled into the lower respiratory tract, and the values were 19.9 U and 49.9% U/U for ACL0, then significantly increased to 35.6 U and 82.1% U/U for ACL15, and further enlarged to >45.2 U and >88.5% U/U for ACL30 and ACL45. In addition, the FPD_<1.66_ and FPF_<1.66_ signified the amount and fraction of NSD powders that can be inhaled into the alveolar regions. Unsurprisingly, the FPD_<1.66_ and FPF_<1.66_were only 7.4 U and 18.7% U/U for ACL0, while significantly increased to 14.4 U and 33.1% U/U for ACL15, then further enlarged to 21.1 U and 40.8% U/U for ACL30 and 22.4 U and 43.9% U/U for ACL45. These data confirmed that the presence of leucine in formulations can greatly increase the amount and fraction of fine particles and extra-fine particles and indicated that the dispersibility of NSD powders was enhanced to such a high level of predominately mini aggregates and largely single particles. These in vitro deposition data of the NSD powders were entirely in agreement with their physical size measurements (Figures 1 and 2), hence statistically verified the strong capability of leucine to enhance the dispersibility of nanoparticle-based dry powders by reducing the surface energy, thus diminishing interparticle cohesion.

**Table 2.** In vitro aerosolization performance of NSD powders.

NSD powders	TD (U)	RD (U)	RF (%, U/U)	ED (U)	EF (%, U/U)	ID (U)	IF (%, U/U)	FPD_<4.46_ (U)	FPF_<4.46_ (%, U/U)	FPD_<1.66_ (U)	FPF_<1.66_ (%, U/U)
ACL0	70.0	57.9(1.3)^I^	82.7(1.8)^I^	52.3(0.8)^I^	90.2(0.7)^I^	39.8(0.4)^I^	68.7(2.2)^I^	19.9(1.5)^I^	49.9(3.2)^I^	7.4(0.7)^I^	18.7(1.6)^I^
ACL15	70.0	58.8(1.9)^I^	84.0(2.7)^I^	52.7(1.3)^I^	89.7(0.9)^I^	43.4(0.8)^II^	73.7(2.3)^II^	35.6(2.1)^II^	82.1(2.0)^II^	14.4(1.6)^II^	33.1(1.5)^II^
ACL30	70.0	61.3(1.8)^I^	87.6(2.8)^I^	58.0(2.3)^II^	94.6(0.7)^II^	51.7(2.8)^III^	84.3(1.9)^III^	45.8(2.5)^III^	88.5(1.4)^III^	21.1(1.6)^III^	40.8(0.9)^III^
ACL45	70.0	61.2(1.7)^I^	87.4(2.6)^I^	54.6(2.1)^I,II^	89.1(0.8)^I^	51.1(2.5)^III^	83.5(1.9)^III^	45.5(2.8)^III^	88.9(1.3)^III^	22.4(1.6)^III^	43.9(0.7)^IV^

ED: Collected from throat to MOC; EF: ED/RD; ID: Collected from S1 to MOC; IF: ID/ED.

FPD_<4.46_: Collected from S3 to MOC; FPF_<4.46_: FPD_<4.46_/ID; FPD_<1.66_: Collected from S5 to MOC; FPF_<1.66_: FPD_<1.66_/ID.

The in vitro deposition of NSD powders was determined by NGI with a pre-separator at a flow rate of 60 L/min for 4 s under the relative humidity of 35%. After deposition, the powders were collected from all parts, including the capsules, device, throat, pre-separator, stages 1–7, and MOC in use of 10 mL of diethanolamine–HCl (1 M, pH 9.8, 0.5 mM MgCl_2_) buffer, respectively. The AKP activities in each sample were quantified colorimetrically via a catalysis reaction on the substrate. The TD, RD, RF, ED, EF, ID, IF, FPD**_<4.46_**, FPF**_<4.46_**, FPD**_<1.66_**, and FPF**_<1._**_66_ were subsequently calculated to reveal the actual powder deposition in NGI. Each metric was compared in statistics, and the different Roman numbers (i.e. I, II, III, and IV) in the same column represented significant differences among the values. The data are expressed as mean ± S.D., *n *= 3.

### 3.4. Calculations of derived metrics based on USP Chapter <601>

**Figure 4.**
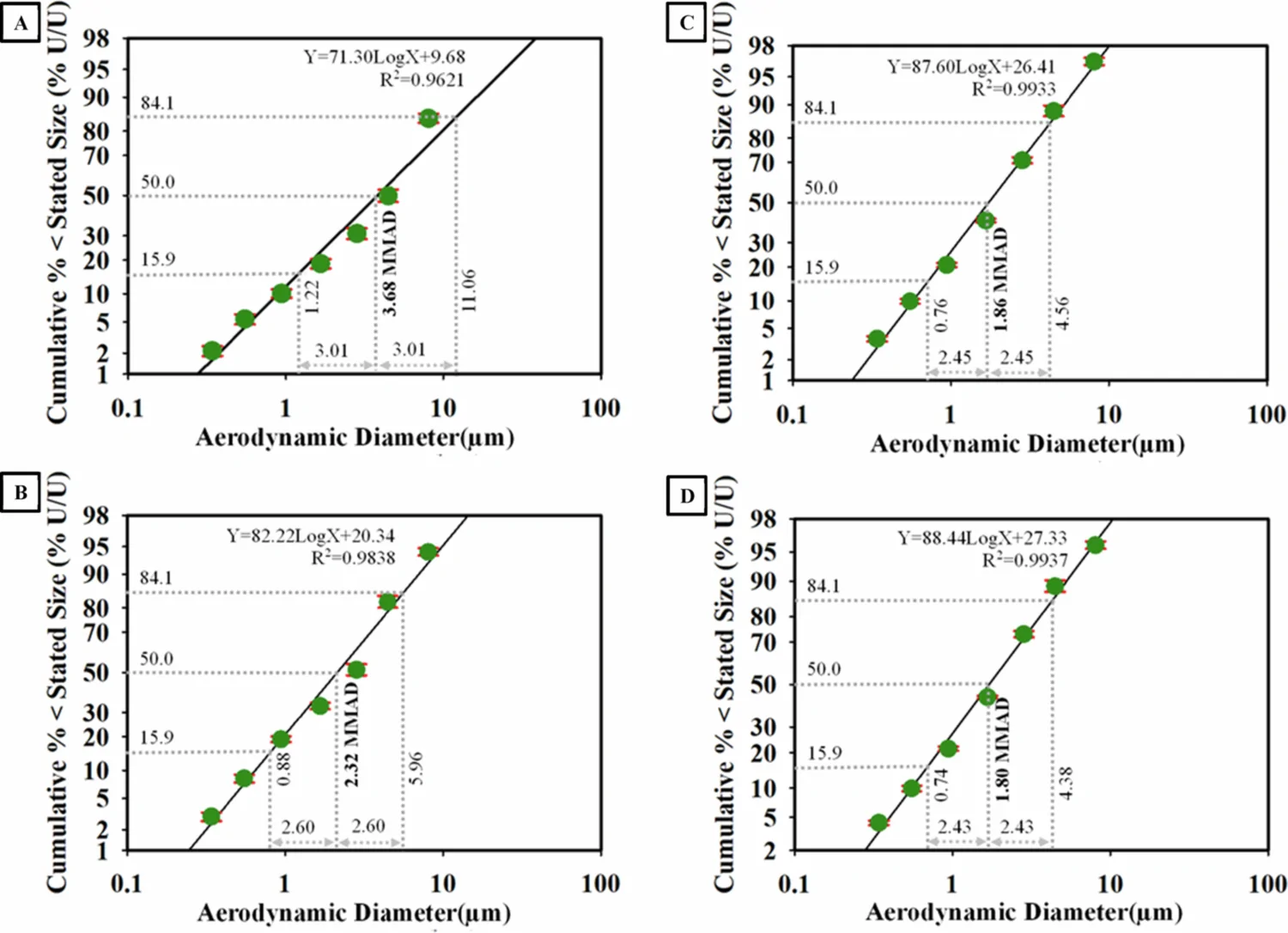
The cumulative APSDs for NSD powder aerosols were constructed following Chapter <601>. (A) ACL0; (B) ACL15; (C) ACL30; (D) ACL45. For the construction of a cumulative APSD under the guidance of Chapter <601>, the cumulative percentages of AKP less than the stated aerodynamic sizes were plotted in probability scale on the ordinate, as a function of the logarithm of the aerodynamic sizes in diameter on the abscissa. Seven points were plotted from stage 7 to stage 1, and the eighth point was out of range in probability scale, as it represented a cumulative percentage of 100% less than 12.8 µm versus the aerodynamic size of 12.8 µm. The linear regression equation was then generated as the theoretical model to represent the cumulative APSD (*p* < 0.05). Based on this linear regression model, the fitted values were calculated for derived metrics of FPF_<5,<601>_, FPD_<5,<601>_, FPF_<2,<601>_, and FPD_<2,<601>_, MMAD_<601>_, GSD_<601>_.

For the entire evaluation of the APSD of an aerosol, the MMAD and GSD are mandatory to be determined in regulation. Besides, other critical derived metrics are also required, including FPD_<5_, FPF_<5_, FPD_<2_, and FPF_<2_, as they suggest powder deposition in the lower respiratory tract and in alveolar regions, work as pivotal attributes for quality control, and are indicators for targeted drug delivery to the lungs in clinical trials. The evaluation of these derived metrics relies on the cumulative APSD. For the construction of the cumulative APSD, Chapter <601> states that the cumulative percentages of AKP amount less than the stated cut-off aerodynamic diameters are plotted on a probability scale on the ordinate against the logarithm of the aerodynamic sizes on the abscissa to produce a linear regression equation that works as a theoretical model to define the cumulative APSD for the calculation of the derived metrics. As shown in Figure 4, for each NSD powder, a linear regression equation was generated as the statistical model to describe the cumulative APSD, and the coefficient of determination (R^2^) was shown to represent how well the theoretical model could explain the experimental data – a higher value suggesting a greater level of goodness-of-fit. For ACL0, the linear regression equation was Y = 71.30LogX + 9.68, with R^2^ of 0.9621 as the lowest (Figure 4A), suggesting the poorest correlation of the theoretical model to experimental data. ACL15 had a cumulative APSD following Y = 82.22LogX + 20.34, and the larger value (0.9838) of R^2^ suggested a greater goodness-of-fit of the model to the investigational figures (Figure 4B). The cumulative APSDs for ACL30 and ACL45 followed linear regression models of Y = 87.60LogX + 26.41 (R^2^ = 0.9933) (Figure 4C) and Y = 88.44LogX + 27.33 (R^2^ = 0.9937) (Figure 4D), respectively. The highest R^2^ values confirmed the exceptional goodness-of-fit of these materials to the experimental facts. Additionally, the slope was continuously increased from ACL0 to ACL45, suggesting a constantly improved uniformity of the particle size distribution with reduced ‘scatters’ of particles around the median (Kottler 1950; Mort 2023).

Based on these theoretical models, the critical derived metrics for the APSDs were calculated and are listed in Table 3. Clearly, the FPD_<5,<601>_ and FPF_<5,<601>_, representing the powders that can be inhaled into the lower respiratory tract, were individually of 23.7 U and 59.5% U/U for ACL0, whereas significantly increased to 33.7 U and 77.8% U/U for ACL15, and additionally augmented to 45.3 U and 87.6% U/U for ACL30, then further improved to 45.6 U and 89.1% U/U for ACL45. In addition, the FPD_<2,<601>_ and FPF_<2,<601>,_ signifying powders that can be inhaled into alveolar regions, were separately 12.4 U and 31.1% U/U for ACL0, then gradually increased to 19.6 U and 45.1% U/U for ACL15, 27.3 U and 52.8% U/U for ACL30, and 27.6 U and 54.0% U/U for ACL45, respectively. Furthermore, for completely describing APSD, the MMAD and GSD were subsequently calculated, with the values of 3.68 µm and 3.01 for ACL0, afterward progressively reduced to 2.29 µm and 2.60 for ACL15, further to 1.86 µm and 2.45 for ACL30, and eventually to 1.80 µm and 2.43 for ACL45. These data were perfectly agreed with the physical size distributions of NSD powders (Figure 2), where the NSD powders turned to have decreased modes and contracted Spans as the increase of leucine in formulations, suggesting enhanced dispersibility and reduced interparticle cohesiveness among the nanoparticle-based NSD powders.

**Table 3.** Fitted values for derived metrics calculated via linear regression models as regulated in Chapter <601>.

NSD powders	FPD_<5, <601>_ (U)	FPF_<5, <601>_ (%, U/U)	FPD_<2, <601>_ (U)	FPF_<2, <601>_ (%, U/U)	MMAD_<601>_ (µm)	GSD_<601>_
ACL0	23.7	59.5	12.4	31.1	3.68	3.01
ACL15	33.7	77.8	19.6	45.1	2.29	2.60
ACL30	45.3	87.6	27.3	52.8	1.86	2.45
ACL45	45.6	89.1	27.6	54.0	1.80	2.43

According to Chapter <601>, the cumulative percentages of AKP amount less than the stated cut-off aerodynamic diameters were plotted in probability scale on the ordinate, as a function of the logarithm of aerodynamic diameters on the abscissa. The linear regression models were statistically produced (as shown in Figure 4) as the cumulative APSD for the calculation of derived metrics of FPF_<5, <601>_, FPD_<5, <601>_, FPF_<2, <601>_, and FPD_<2, <601>_. MMAD _<601>_, GSD _<601>_.

### 3.5. Evaluation of derived metrics based on USP Chapter <1604>

**Figure 5.**
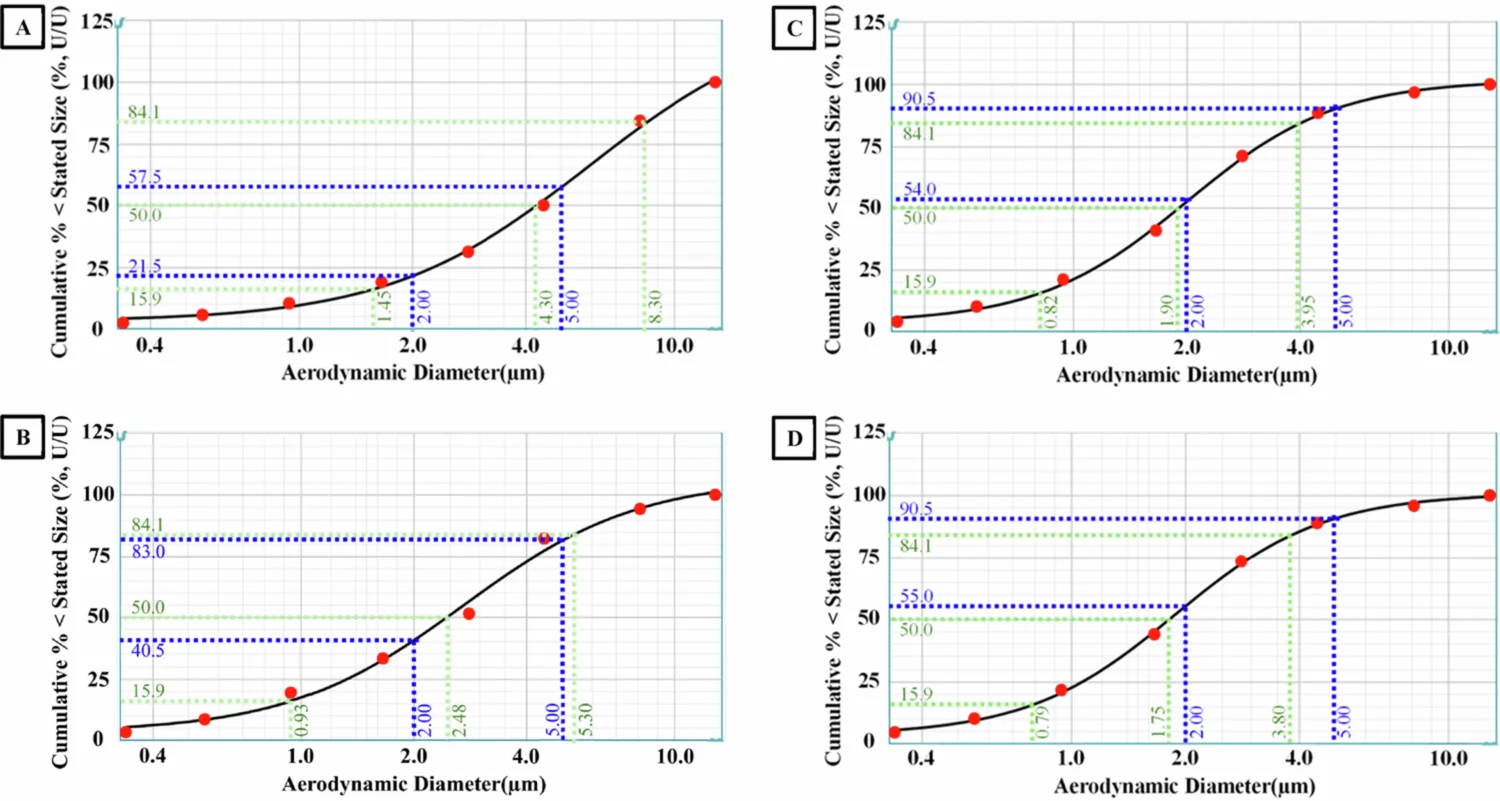
The cumulative APSDs for NSD powder aerosols constructed following Chapter <1604>. (A) ACL0; (B) ACL15; (C) ACL30; (D) ACL45. For the construction of a cumulative APSD under the guidance of Chapter <1604>, the cumulative percentages of AKP less than the stated aerodynamic sizes were plotted in normal scale on the ordinate as a function of the logarithm of aerodynamic sizes in diameter on the abscissa. In this situation, besides the seven points from stage 7 to stage 1, the eighth point at which the cumulative percentage of 100% less than 12.8 µm versus the aerodynamic size of 12.8 µm, can be plotted in normal scale on the ordinate. The best-fit curve was produced through these eight points, representing the cumulative APSD as regulated in Chapter <1604>. Based on this best-fit curve, the actual values were determined for derived metrics of FPF_<5, <1604>_, FPD_<5, <1604>_, FPF_<2, <1604>_, and FPD_<2, <1604>_, MMAD_<1604>_, GSD_<1604>_.

According to the recently published Chapter <1604>, the impactor-sized mass was employed for the construction of the cumulative APSD, and this updated method requires plotting the cumulative mass percentage of powders less than the stated size in normal scale on the ordinate versus the logarithm of the aerodynamic sizes on the abscissa to generate a best-fit curve. Hence, the use of this best-fit curve subsequently produces actual values for FPF_<5,<1604>_, FPD_<5_, _<1604>_, FPF_<2,<1604>_, FPD_<2, <1604>_, MMAD_<1604>_, and GSD_<1604>_ (Figure 5, Table 4). In terms of fine particle doses and fractions, FPD_<5_, _<1604>_ and FPF_<5,<1604>_ were as low as 22.9 U and 57.5% U/U for ACL0, while they were rocketed up to 36.0 U and 83.0% U/U for ACL15, and further augmented to 46–47 U and 90.5% U/U for ACL30/ACL45. Following the comparable tendency, FPD_<2,<1604>_ and FPF_<2,<1604>_ were only 8.6 U and 21.5% U/U for ACL0, but they were approximately doubled to 17.6 U and 40.5% U/U for ACL15 and further increased to ~28 U and 54%–55% U/U for ACL30 and ACL45. Unquestionably, the increase in leucine in the formulation can distinctively improve drug delivery to the lower respiratory tract and peripheral regions, indicating progressively enhanced dispersibility and continuously reduced interparticle cohesion for nanoparticle-based NSD powders. This affirmation can be further supported by the reduced MMADs and shrunk GSDs. The MMAD_<1604>_ and GSD_<1604>_ were at the biggest values of 4.30 µm and 2.39 for ACL0, while they were reduced to 2.48 µm and 2.38 for ACL15, then diminished to 1.90 µm and 2.19 for ACL30, and further lessened to 1.75 µm and 2.19 for ACL45. These data indisputably confirmed the strong ability associated with leucine for reducing interparticle cohesiveness, hence enhancing the dispersibility and aerosolization performance of nanoparticle-based NSD powders, and that NSD powders with higher leucine contents (i.e. ACL30 and ACL45) can be predominately inhaled into the lower respiratory tract with a large population further delivered to the alveolar regions.

**Table 4.** Actual values for derived metrics evaluated from the best-fit curve as regulated in Chapter <1604> .

NSD powders	NSD_<1604>_ (U)	NSF_<1604>_ (%, U/U)	CPD_<1604>_ (U)	CPF_<1604>_ (%, U/U)	FPD_<1604>_ (U)	FPF_<1604>_ (%, U/U)	EPD_<1604>_ (U)	EPF_<1604>_ (%, U/U)	FPD_<5,<1604>_ (U)	FPF_<5,<1604>_ (%, U/U)	FPD_<2,<1604>_ (U)	FPF_<2,<1604>_ (%, U/U)	MMAD_<1604>_ (µm)	GSD_<1604>_
ACL0	12.5(1.2)^I^	23.9(1.9)^I^	19.9(1.1)^I^	38.1(1.5)^I^	15.8(1.0)^I^	30.3(2.5)^I^	4.0(0.4)^I^	7.7(0.9)^I^	22.9	57.5	8.6	21.5	4.30	2.39
ACL15	9.4(0.6)^II^	17.8(1.8)^II^	7.7(0.6)^II^	14.7(1.3)^II^	27.4(2.0)^II^	51.8(2.0)^II^	8.3(1.0)^II^	15.7(1.2)^II^	36.0	83.0	17.6	40.5	2.48	2.38
ACL30	6.3(0.6)^III^	10.8(1.4)^III^	5.9(0.4)^III^	10.3(1.0)^III^	35.0(2.2)^III^	60.4(1.5)^III^	10.8(0.9)^III^	18.5(1.0)^III^	46.8	90.5	27.9	54.0	1.90	2.19
ACL45	3.4(0.5)^IV^	6.3(1.3)^IV^	5.7(0.4)^III^	10.4(1.1)^III^	34.5(2.1)^III^	63.2(1.4)^III^	11.0(1.0)^III^	20.1(1.0)^III^	46.3	90.5	28.1	55.0	1.75	2.19

According to Chapter <1604>, the cumulative percentages of AKP amount less than the stated cut-off aerodynamic diameters were plotted in normal scale on the ordinate, as a function of the logarithm of aerodynamic diameters on the abscissa. The best-fit curve was generated (as shown in Figure 5) for the evaluation of derived metrics of FPF_<5,<1604>_, FPD_<5,<1604>_, FPF_<2,<1604>_, FPD_<2,<1604>_, MMAD_<1604>_, and GSD_<1604>_. Furthermore, for each powder, the deposition data (as shown in Figure 3) were grouped to obtained derived metrics of UD_<1604>_, CPD_<1604>_, FPD_<1604>_, and EPD_<1604>_, and the these values were individually against the ID to produce fractions of UF_<1604>_, CPF_<1604>_, FPF_<1604>_, EPF_<1604>_. The different Roman numbers (i.e. I, II, III, and IV) in the same column represented significant differences among the values. The data are expressed as mean ± S.D., n = 3.

Chapter <1604> also regulates stage groupings of powders based on their aerodynamic sizes, targeted to predict the amount and fractions of aerosolized powders deposited in different regions of the respiratory tract. Based on their deposition stages in in vitro testing, drug particles are separately collected from the throat and pre-separator (D_ae_ > 12.8 µm) as Group 1; stages 1–2 (12.8 µm > D_ae_ > 4.46 µm) as Group 2; stages 3–5 (4.46 µm > D_ae_ > 0.94 µm) as Group 3; and stages 6-MOC (0.94 µm > D_ae_ > 0.14 µm) as Group 4. These four groups are sequentially labeled as NPD/NPF, CPD/CPF, FPD/FPF, and EPD/EPF for additional characterization of an APSD. The values for these derived metrics are listed in Table 4 to further define the APSDs of NSD powder aerosols. Clearly, NPD_<1604>_ and NPF_<1604>_ were as high as 12.5 U and 23.9% U/U for ACL0, while significantly reduced to 9.4 U and 17.8% U/U for ACL15, and additionally diminished to 6.3 U and 10.8% U/U for ACL30, then further lessened to 3.4 U and 6.3% U/U for ACL45. Besides, CPD_<1604>_ and CPF_<1604>_ demonstrated the similar inclination, and they were of 19.9 U and 38.1% U/U for ACL0, then plummeted in significance to 7.7 U and 14.7% U/U for ACL15, and further reduced to ~6 U and ~10% U/U for ACL30 and ACL45. These data clearly demonstrated that the amount of non-sized and coarse particles was the highest for the leucine-free powders, but substantially diminished in the presence of leucine in the formulation and further reduced with the increase in leucine content, confirming the effect of leucine on the de-aggregation of NSD powders. This affirmation can be further supported in another way by the increase of FPD/FPF and EPD/EPF. The FPD_<1604>_ and FPF_<1604>_ were 15.8 U and 30.3% U/U for ACL0, while significantly augmented to 27.4 U and 51.8% U/U for ACL15, and further increased to ~35.0 U and >60% U/U for ACL30 and ACL45. Following the same propensity, EPD_<1604>_ and EPF_<1604>_ were only 4.0 U and 7.7% U/U for ACL0, but significantly increased to 8.3 U and 15.7% for ACL15, and additionally improved to ~11 U and ~18% U/U for ACL30/ACL45. Clearly, these data confirmed that leucine worked competently to enhance the dispersibility of the nanoparticle-based NSD powders and immensely increased the amount and fractions of fine and extra-fine particles used for powder delivery to the lower respiratory tract, particularly the peripheral regions. Overall, these data strongly suggest that the use of leucine in formulations can endow nanoparticle-based NSD powders with exceptional dispersibility and superior aerosolization performance, leading to largely reduced powder deposition in the upper airway and immensely increased drug delivery to deep lungs.

## 4. Discussion

Over many years, dry powders have been a hot topic for the delivery of biologics to the lungs, and this tendency is particularly intensified in current situations, as they are increasingly important for pulmonary delivery of mRNAs that are considered as the next generation of medicines for vaccination and for the treatment of gene-deficiency diseases. In this study, we addressed three critical issues for developing NSD powders of biologics and for determining derived metrics to better describe APSD, including: (1) The protection of heat-sensitive biologics during the harsh NSD process; (2) The improvement of dispersibility and aerosolization performance for nanoparticle-based NSD powders; and (3) The employment of traditional Chapter <601>  and newly published Chapter <1604>  to evaluate and compare derived metrics, on which, no studies have implemented these two Chapters in a real situation and compared their differences in handling APSD data and in acquired values of derived metrics.

### 4.1. General discussion on AKP protection and physicochemical properties of NSD powders

In this study, cyclodextrin was utilized as a thermo-protectant for AKP during spray-drying. This is because biologics require additional forces to support their 3D structures for the maintenance of biological functions, and this can be achieved through the formation of hydrogen bonds with the medium into which they are dispersed (Shire 2009). In aqueous solutions, water molecules provide hydrogen bonding to biologics for this purpose. In the dry state, excipients are needed as substitutes for water removal to offer hydrogen bonding for stabilizing biologics. General excipients that have been proven to be effective consist of disaccharides (i.e. lactose and trehalose) (Li et al. 2003, Li et al. 2005a, 2005b) and polysaccharides (i.e. chitosan and cellulose) (Li and Birchall 2006; Li et al. 2010; Li and Seville 2010). In this study, we further confirmed that the oligosaccharide of cyclodextrin can also effectively protect enzymes during the spray-drying process, and that over 94% w/w of thermolabile AKPs can maintain their biological functions in dry powders, implying that their tertiary/quaternary structures are well preserved during the spray-drying process. Therefore, our studies have proven that saccharides are exceptional thermo-protectants, and di-, oligo-, and poly-saccharides can effectively shield proteins and gene materials from heat, thereby preserving their 3D structures and biological functions during the spray-drying process.

Additionally, this study also demonstrated that the NSD yields were of about 48%–51% w/w, which seemed lower than those (~90% w/w) reported somewhere else (Bürki et al. 2011). A possible reason for this might be related to the very small amount of formulations used for spray drying, so that, even a very thin layer of NSD powder was attached to the large inner surface of the collection chamber, which could still possess a large fraction of the total mass. Hence, with an increase in feed for spray-drying, the yields of NSD powders can be expected to increase.

Furthermore, this study showed that cyclodextrin-based NSD powders (ACL0) had a moisture content of approximately 6% w/w, and this value was greater than that (3% w/w) for disaccharide (i.e. lactose and trehalose) – based (Xu et al. 2014) but less than those (7%–10% w/w) for polysaccharide (i.e. chitosan, cellulose, and starch) – based (Li et al. 2010; Li and Seville 2010; Jiang et al. 2017) spray-dried powders. These data suggest that, as the saccharide chains elongate, their use as excipients increases the moisture content of spray-dried powders. This is not surprising, as the extension of saccharide chains would result in the formation of colloids in water, so that water molecules are trapped inside the colloids and cannot be evaporated during spray-drying (Yu et al. 2019; Li and Xu 2023), which consequently causes an increase in the moisture content of spray-dried powders and further affects their aerosolization performance. Fortunately, the moisture content can be reduced by the addition of leucine, and this propensity is reinforced by increasing its fraction in the formulation. This is due to the hydrophobic nature of leucine; hence, water molecules can be more readily evaporated from droplets during the spray-drying process, leading to a decrease in the moisture content of spray-dried powders, which facilitates powder flowability, dispersibility, and aerosolization performance (Cui et al. 2018; Li and Xu 2023; Zhang et al. 2024).

### 4.2. The improvement of aerosolization performance for NSD powders

NSD powders are predominately of 0.5–2 µm, which are ideal for drug delivery to deep lungs through inhalation. However, the particles in this size range have immensely increased surface areas and augmented surface energy, which causes NSD particles to have enlarged cohesiveness, severe aggregation, and poor aerosolization performance. To overcome these problems, it is necessary to modify the components of NSD powders for the improvement of dispersibility, aerosolization behavior, and lung deposition. Our previous study showed that the use of cyclodextrin can increase the surface roughness of spray-dried powders, hence reduce interparticle contact, enhance powder dispersibility, and improve aerosolization manners (Li et al. 2005b), which supported the following studies in other research groups and was additionally confirmed by their data (Cabral-Marques and Almeida 2009; Dufour et al. 2015). Therefore, cyclodextrin was selected as the base material for the preparation of NSD powders in this study. Indeed, the cyclodextrin-based NSD powders had satisfactory aerosolization performance, with an EF of 90.2% U/U, IF of 68.7% w/w, and FPF_<4.46_ of 49.9% U/U, confirming that cyclodextrin performed well in bestowing NSD powders with good flowability and dispersibility. However, the FPF_<1.66_ was only 18.7% U/U, which was below the expected level in terms of their physical sizes, primarily < 2 µm and largely < 1 µm – representing an ideal size for being inhaled into alveolar regions. Therefore, these in vitro deposition data indicated that cyclodextrin-based NSD particles were extensively aggregated to form agglomerates of approximately 2–5 particles (MMAD_<1604>_ = 4.30 µm), as estimated in the inhalation airflow, so that a large portion of the powder in the form of agglomerates could still be inhaled into the lower respiratory tract, but only a small fraction of powders were still isolated as single particles that could be further conveyed to the alveolar regions.

To further improve the aerosolization performance of the spray-dried powders, it is necessary to enhance their flowability and dispersibility. Leucine is a well-known dispersibility enhancer that has been well studied (Li et al. 2003; Alhajj et al. 2021; Dieplinger et al. 2024), and this is because of its unique structure. Leucine has amino and carboxylic groups that are hydrophilic and a side chain of the isobutyl group that is hydrophobic, so that leucine can be dissolved in water but has the nature of repelling water molecules, hence it is classified as a hydrophobic amino acid. The hydrophobic nature of leucine facilitates the quick escape of water molecules from the droplets, which subsequently causes it to be saturated and precipitated at an earlier time during the spray-drying process. The precipitated leucine molecules hence form an outer layer surrounding the core of the droplet, leading to the generation of spray-dried particles enriched with leucine molecules on the surface (Feng et al. 2011). The abundance of leucine molecules on the surface has been reported to be able to diminish surface energy (Lechuga-Ballesteros et al. 2008), which consequently weakens interparticle cohesion, improves aerosolization performance, and resists moisture invasion (Li et al. 2016; Cui et al. 2018). Additionally, the addition of leucine to the formulation can augment the surface roughness, such as the generation of golf-ball shape associated with leucine-modified spray-dried particles, as confirmed in this study and in our previous report (Li et al. 2003). An increase of surface roughness can subsequently reduce the possibilities of contact among spray-dried particles, henceforward further improving their dispersibility and lung deposition. The data reported in this study exactly match these principles. The addition of leucine into formulations can noticeably increase the dispersibility of NSD powders, and this propensity is intensified as the increase of its mass fraction, as evidenced by FPF_<1.66_ (indicating the portion of individual particles in airflow) massively augmented from 18.7% U/U for leucine-free ACL0 to 33.1% U/U for ACL15 and further to 41%–43% U/U for ACL30/ACL45. These figures clearly demonstrate that the dispersibility of NSD powders can be distinctively improved by the addition of leucine, and this propensity can be further strengthened with its extra amount in formulations, strongly supporting its powerful capability for diminishing cohesiveness and enhancing the dispersibility of particles even at the nanoscale.

### 4.3. The comparisons of derived metrics determined by chapters <601> and <1604>

APSD is a critical quality attribute for OIPs in regulation, which depicts the size distribution of particles or droplets in an aerosol according to their aerodynamic sizes in diameter. The APSD is determined by multistage cascade impactors, and NGI is the most advanced device with full resolution for characterization, hence it has been widely employed in the inhalation sector. After drug deposition in the NGI, the amounts of drug impacted in all the components are individually quantified and plotted against impactor stages to produce the deposition pattern. Subsequently, the mass percentages of the components are calculated for the construction of the cumulative APSD, which is an essential prerequisite for determining the derived metrics. Two USP Chapters, the traditional <601>  (USP <601> 2023a) and recently published <1604>  (USP <1604> 2023b), regulate the approaches for handling APSD data to construct a cumulative APSD henceforward to determine the derived metrics. However, distinctive differences exist between these two Chapters, which are summarized below. (1) The total mass can be different for the calculation of mass percentages of components. In Chapter <601> , for NGI with (Apparatus 5) or without (Apparatus 6) the pre-separator, the mass deposited onto NGI components of stages 1–7 and MOC are all and always summated as the total mass for the calculation of mass percentage for each component, hereafter to acquire cumulative mass percentages less than the stated sizes in aerodynamic diameter for the construction of cumulative APSD. In Chapter <1604>, only the impactor-sized mass is accountable for the calculation of total mass, which hence has no difference from Chapter <601> for Apparatus 5 (with pre-separator, S1 sized), while only referring to the overall mass collected from stage 2 to MOC for Apparatus 6 (no pre-separator, S1 non-sized). (2) Different approaches are employed to generate the correlation between the cumulative mass percentages less than the stated sizes and the aerodynamic sizes in diameter. In Chapter <601> , as regulated, the cumulative percentages of mass less than the stated sizes in probability scale on the ordinate are plotted as a function of the logarithm of the aerodynamic sizes in diameter on the abscissa. A linear regression equation is then generated with R^2^ presented to show the level of goodness-of-fit between the linear model and experimental data. Based on this linear regression equation, the derived metrics of FPF_<5_ and FPF_<2_ can be determined, and the parameters of D_15.9_, D_50_, and D_84.1_ can then be calculated for the evaluation of MMAD (=D_50_) and GSD (=[D_84.1_/D_15.9_]^1/2^). In Chapter <1604> , it is necessary to plot the cumulative mass percentages less than the stated sizes in normal scale on the ordinate against the logarithm of aerodynamic sizes in diameter on the abscissa; then, a best-fit curve is generated to show their finest correlation. This best-fit curve is subsequently employed to evaluate the derived metrics of FPF_<5_, FPF_<2_, MMAD (=D_50_), and GSD (=[D_84.1_/D_15.9_]^1/2^). (3) The stage groupings of the deposition data are regulated in Chapter <1604> . The stage groupings of deposition data have not been structured in Chapter <601>  but have become indispensable derived metrics for additionally describing APSD in Chapter <1604> . This concept and practice are introduced for quality control purposes and may also be indicators for regional depositions in human airways, which refer to the consolidation of mass deposition data into ‘non-overlapping groups to summarize the distribution of the mass of drug substance emitted from the inhaler’ (USP <1604> 2023b). These groups are defined as follows: Group 1, NSD/NSF, representing drug deposition in the oral cavity; Group 2, CPD/CPF, signifying drug deposition in the pharynx/larynx; Group 3, FPD/FPF, suggesting drug deposition in the lower respiratory tract; and Group 4, EPD/EPF, predicting drug deposition in alveolar regions. At the flowrate of 60 L/min for NGI with pre-separator, the particle size ranges in aerodynamic diameter for these Groups are non-sized for Group 1 (induction port and pre-separator), 12.8–4.46 µm for Group 2 (stages 1-2), 4.46–0.94 µm for Group 3 (stages 3–5), and 0.94–0.14 µm for Group 4 (stages 6-MOC).

In this study, the pre-separator was connected to the NGI and capped an upper size limit (12.8 µm) for stage 1, so that all mass impacted in NGI stages was sized and hence summated as the total mass for the calculation of cumulative APSD. Following Chapter <601> , the cumulative mass percentages less than the stated sizes were plotted on a probability scale on the ordinate against the logarithm of aerodynamic sizes in diameter on the abscissa to produce a linear regression equation (Figure 4). It should be noted that the R^2^ was the lowest for ACL0, while progressively increased to >0.99 for ACL45, suggesting an improvement in goodness-of-fit between the theoretical model and experimental data. Based on linear regression equations, the derived metrics were calculated to produce the fitted values. In Chapter <1604> , the cumulative mass percentages less than the stated sizes were plotted on a normal scale on the ordinate as a function of the logarithm of the aerodynamic sizes in diameter on the abscissa to produce a best-fit curve (Figure 5). Based on this best-fit curve, the derived metrics were evaluated to generate actual values.

**Figure 6.**
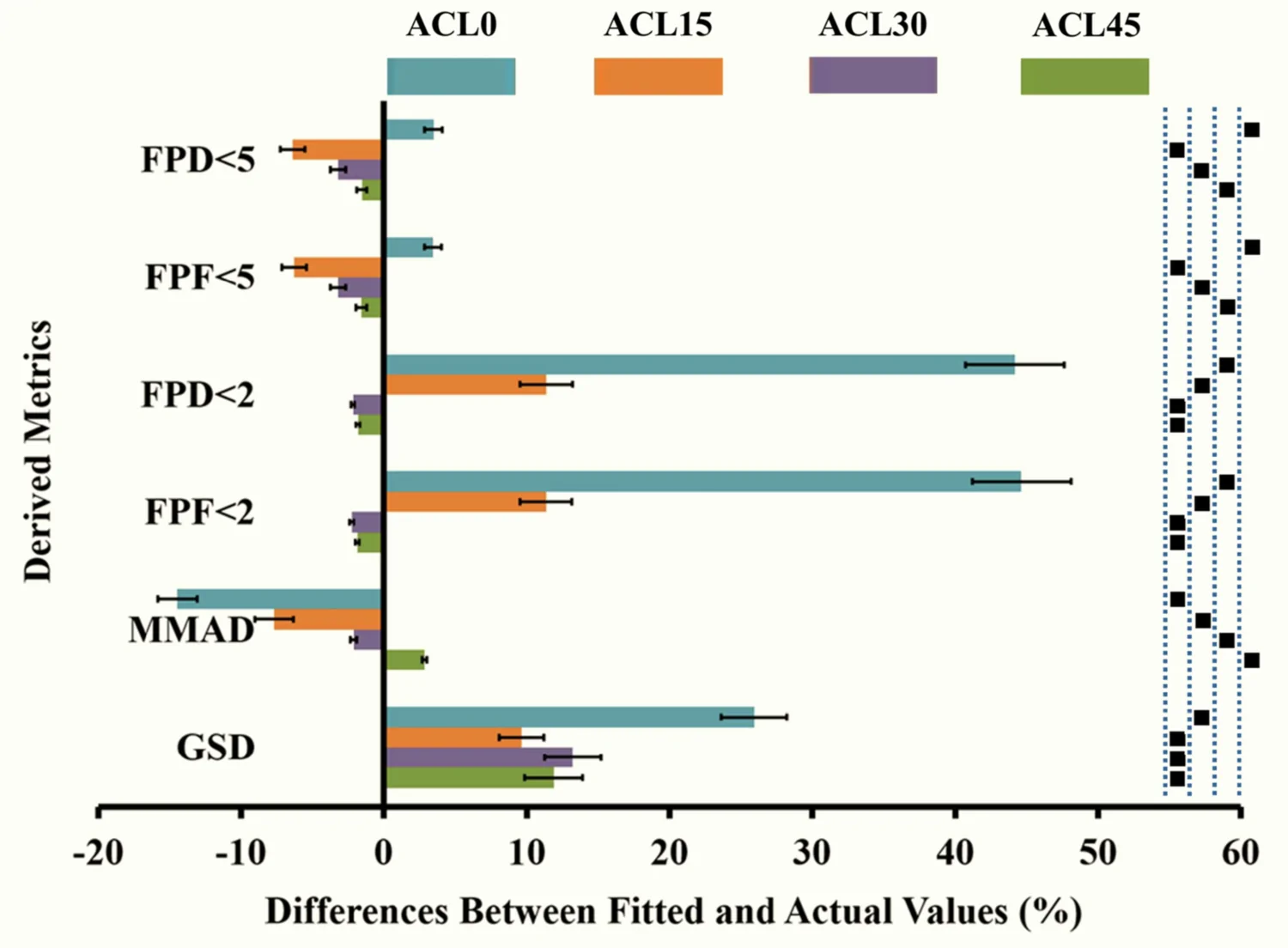
The differences between fitted values and actual values for derived metrics. For a specific derived metric, the difference between fitted value (V_<601>_, calculated by linear model in Chapter <601>) and actual value (V_<1604>_, evaluated by best-fit curve in Chapter <1604>) was calculated using the formula of (V_<601>_ − V_<1604>_) ∗ 100/V_<1604>_. The less difference suggests a greater authenticity for the fitted value. In terms of a specific derived metric, the differences for all NSD powders were statistically compared by one-way ANOVA (Duncan’s test, *p* < 0.05), and the sign of black solid squares on different vertical dotted lines represented significant differences. Data were expressed as mean ± S.D., *n* = 3.

To symbolize the difference between these two values determined by Chapters <601> and <1604> for the same derived metric, a comparison of (Value_<601>_ − Value_<1604>_) ∗ 100/Value_<1604>_ was then made to disclose the change in the fitted value to the actual value. As shown in Figure 6, it was clearly demonstrated that ACL0 had the greatest changes on most derived metrics, with alteration values of 45% for FPD_<2_/FPF_<2_, 14% (negative) for MMAD, 25% for GSD; 3% for FPD_<5_/FPF_<5_. In comparison, ACL15 had significantly decreased but still large differences in fitted values for derived metrics, with 11% for FPD_<2_/FPF_<2_, 8% (negative) for MMAD, 10% for GSD, and 6% (negative) for FPD_<5_/FPF_<5_. ACL30 had the fitted values largely comparable to the actual values, with a variation of only 3% (negative) for FPD_<5_/FPF_<5_, 2% (negative) for FPD_<2_/FPF_<2_, 2% (negative) for MMAD, and 13% for GSD. ACL45 displayed negligible differences (<2% or less) between the fitted values and actual values for most of the derived metrics, except for (12%) for the GSD. In comparison with ACL0 and ACL15, ACL30 and particularly ACL45 showed significantly reduced differences between the fitted values and the actual values for most of the derived metrics. Therefore, from ACL0 to ACL45, as the increase of leucine in the formulation, the NSD powder aerosol had a cumulative APSD from which the acquired derived metrics had a lower difference between the fitted values calculated by the linear regression model as normalized in Chapter <601>  and the actual values evaluated by the best-fit curve as regulated in Chapter <1604>.

An insight can be generated by linking these data to APSDs for NSD powder aerosols. Although all NSD powder aerosols demonstrated a unimodal APSD (Figure 3), the mode of the APSD for each individual powder aerosol was very different, at stage 2 for ACL0, at stage 3 for ACL15, and at stage 4 for ACL30/ACL45, which subsequently caused the APSD to be long-tailed for ACL0 and ACL15 but of near-perfect symmetry for ACL30 and ACL45 across all sized impactor stages. This transition of the APSD mode from a deviated position to the center of the NGI stages would subsequently improve the level of goodness-of-fit for the linear regression equation. This assertion was distinctly supported by the data shown in Figure 4. For ACL0, the mode of APSD at stage 2 with a large mass portion in stage 2 caused jumped values of cumulative mass percentage less than 8.06 µm, which made the linear regression equation poorly match the experimental data (Figure 4A). Unsurprisingly, this caused the coefficient of determination (R^2^) to be the lowest, leading to the poorest correlation between the fitted values (Figure 4A, Table 3) and actual values (Figure 5A, Table 4), and resulting in the largest changes between them (Figure 6). For ACL15, the mode of APSD at stage 3 instigated a clearly enlarged value of cumulative mass percentage to less than 4.46 µm, hence caused the cumulative mass percentages to be less than 2.82 µm and 1.66 µm lower than the values as anticipated from the model (Figure 4B). Fortunately, ACL15 had much improved goodness-of-fit with most of the experimental data on the line, hence matching the model, leading to an increased coefficient of determination and reduced deviations between the fitted values (Figure 4B, Table 3) and actual values (Figure 5B, Table 4) for the derived metrics (Figure 6). For ACL30 and ACL45, the modes were centered at stage 4 to endow their APSDs with a near-perfect symmetrical shape across all-sized impactor stages. Hence, the cumulative APSDs displayed a near-perfect linear model with R^2^ > 0.99 (Figure 4C and D), leading to the minimal differences or even perfect matches between fitted values (Table 3) and actual values (Figure 5C and D, Table 4) for derived metrics (Figure 6). Therefore, for aerosols that had a unimodal APSD with the mode at the center of the sized impactor stages, the fitted values for the derived metrics calculated following the linear models, as normalized in Chapter <601> are very comparable and have negligible differences from the actual values evaluated from the best-fit curve as regulated in Chapter <1604>. However, for the powder aerosol that displays an APSD with the mode that is largely deviated from the center of the sized impactor stages, the fitted values for the derived metrics can be immensely different from their actual values. In such situations, the use of Chapter <601> for the determination of derived metrics would yield incorrect values, improper specifications for product quality, and erroneous indicators for clinical trials. Hence, it is recommended to follow Chapter <1604> to generate a best-fit curve as the cumulative APSD to determine authentic values for derived metrics.

Chapter <601> is still widely followed by the industry sector for the determination of OIPs on their APSDs and derived metrics, particularly FPD_<5_/FPF_<5_, FPD_<2_/FPF_<2_, MMAD, and GSD, as they are essential parameters to describe the APSD, provide pivotal information for pulmonary drug delivery, and are also indicators for supporting clinical trials. In the current situation, the industry predominately utilizes software to determine these derived metrics, and many product developers may not well understand the working rationale behind them; hence, a high risk exists to produce large errors on these critical quality attributes, which may endanger health and safety. To determine accurate values for these derived metrics following Chapter <601>, the APSD should be unimodal and normal across-sized impactor stages. Despite an unimodal APSD, the shift of mode to a side stage of the impactor will cause the derived metrics to have fitted values calculated by the theoretical model as normalized in Chapter <601>, far away from their actual values as evaluated by the best-fit curve as regulated in Chapter <1604>. To the best of our knowledge, this is the first time to disclose that, under the umbrella of Chapter <601>, the authenticity of fitted values for derived metrics is determined by the APSD profiles and secured only by a unimodal and normal distribution across-sized impactor stages. The data reported in this study would then be able to support researchers and developers in this field to deeply understand the construction of accumulative APSDs under different regulations, and are inspired to increase the accuracy of the analysis of these essential derived metrics, henceforward to make continuous improvements for the quality control of OIPs.

## 5. Conclusion

This study successfully prepared NSD powders with preserved biological activity and high dispersibility, which showed a great potential to efficiently deliver proteins to the targeted regions of the lungs. We confirmed that cyclodextrin can effectively protect proteins from heat attack with > 94% of their biological activities retained after being spray-dried, and verified that the use of cyclodextrin can additionally endow NSD powders largely at a nanosized scale with satisfactory flowability and dispersibility. Further, we extended the capability of leucine to enhance the powder dispersibility and aerosolization behavior of those particles at nanosized ranges and demonstrated that its capability was intensified with an increase in the content of the formulation. Finally, we disclosed for the first time that the authenticity of the derived metrics as calculated following Chapter <601> was determined by the profiles of APSD and secured only when the APSD was unimodal and normal across all-sized impactor stages; hence, the acquired fitted values for the derived metrics had minimal difference from the actual values as determined in Chapter <1604>. The data reported in this study would be able to trigger insights into the increase in data accuracy for APSD analysis and support continuous improvements in inhaled product qualities.

## Data Availability

Data will be made available from the corresponding author on request.
